# Surgical treatment of primary trapezio-metacarpal osteoarthritis by trapeziectomy and ligament reconstruction without tendon interposition. Long-term results of 50 cases

**DOI:** 10.1186/s10195-019-0532-4

**Published:** 2019-07-02

**Authors:** Fernando De Maio, Pasquale Farsetti, Vito Potenza, Lidio Petrungaro, Martina Marsiolo, Alessandro Caterini

**Affiliations:** Department of Orthopedics, University of Rome “Tor Vergata”, Viale Oxford, 81, 00133 Rome, Italy

**Keywords:** Thumb, Trapezio-metacarpal osteoarthritis, Trapeziectomy

## Abstract

**Background:**

Primary trapezio-metacarpal osteoarthritis can be painful and disabling. Surgical treatment is used when conservative treatment, such as splinting or oral analgesics, fails. The purpose of this study was to report the long-term outcomes obtained in 40 patients (50 thumbs) surgically treated for thumb osteoarthritis by trapeziectomy and ligament reconstruction without tendon interposition.

**Materials and methods:**

Forty patients (50 thumbs), with severe trapezio-metacarpal osteoarthritis, surgically treated by trapeziectomy and ligament reconstruction without tendon interposition were reviewed after an average follow-up of 8 years. All patients were women. At follow-up, clinical results were evaluated on the basis of the DASH score, possible presence of pain and the following criteria: palmar abduction of the thumb, carpometacarpal joint opposition of the thumb (Kapandji), extension of the metacarpophalangeal joint and strength of the hand.

**Results:**

The DASH score improved from 42.65 (preoperatively) to 16 (at follow-up), and most patients were asymptomatic. Palmar abduction of the thumb averaged 57 mm. Carpometacarpal joint opposition averaged 8.8. Metacarpophalangeal extension was abnormally increased in 86% of the cases. The strength of the operated hand was comparable to the contralateral side in 46 cases. Radiographic examinations showed a slight proximal migration of the first metacarpal bone (< 3 mm) in all cases but mild signs of carpometacarpal osteoarthritis in only 4 cases.

**Conclusions:**

Based on the reported experience, we believe that primary trapezio-metacarpal osteoarthritis surgically treated by trapeziectomy and ligament reconstruction without tendon interposition allows good long-term results.

**Level of evidence:**

Therapeutic IV

## Introduction

Primary trapezio-metacarpal osteoarthritis is very common, especially in postmenopausal women, and is often complicated by pain and disability of the thumb. Surgical treatment is indicated when conservative treatment fails [1–3].

The more commonly performed procedures for the treatment of trapezio-metacarpal osteoarthritis are: trapezio-metacarpal arthrodesis, trapeziectomy, trapeziectomy with tendon interposition, trapeziectomy with reconstruction of the first intermetacarpal ligament, trapeziectomy with ligament reconstruction and tendon interposition, trapezio-metacarpal arthroplasty, resection of damaged articular surface and interposition of soft tissue, and trapezio-metacarpal joint replacement. However, it is still controversial as to whether one surgical procedure is superior to another in terms of pain, physical function and radiographic imaging [4–8]. Moreover, to the best of our knowledge, few long-term follow-up studies have been published on this subject [1, 2, 7–9].

In this study, we report the long-term results obtained in 40 patients (50 thumbs) surgically treated for painful osteoarthritis of the trapezio-metacarpal joint by the Altissimi surgical technique that consists of excision of the trapezium and reconstruction of the first intermetacarpal ligament by using the distally based half of the flexor carpi radialis tendon, without tendon interposition [7, 10–13].

## Materials and methods

Forty patients (50 thumbs) surgically treated for trapezio- metacarpal arthritis according to Altissimi [13] were reviewed with an average follow-up of 8 years, ranging from 5 to 12 years.

**Fig. 1:**
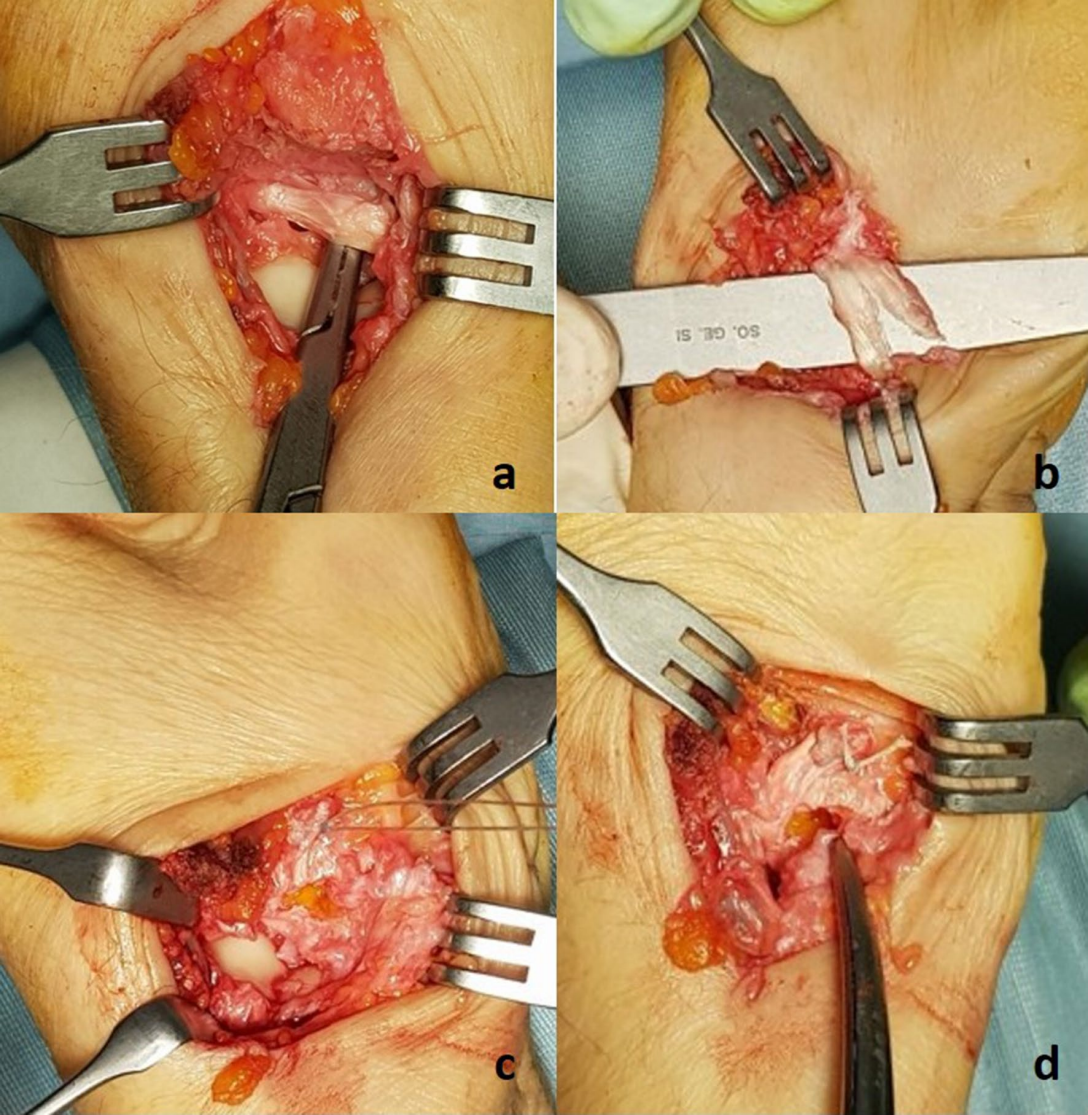
**a** The flexor carpi radialis tendon is found on the bottom of the surgical approach after trapeziectomy. **b** A distally pedicled tendon graft is obtained by splitting the tendon lengthwise into two parts. **c**, **d** The tendon graft is inserted into the base of the first metacarpal to avoid its proximal migration, using a mini anchor

The operation was performed under peripheral anesthesia, using an above-elbow tourniquet. An incision was made along the radial border of the first metacarpal, after which the entire trapezium was excised. A distally pedicled tendon graft, approximately 3.5 cm in length and consisting of the radial half of the flexor carpi radialis (FCR) tendon, was inserted into the dorsal side of the first metacarpal base, at 1 o’clock or 11 o’clock, depending on the right or left metacarpus, respectively, and fixed using a mini anchor. The distal insertion of the half tendon was left intact at the base of the second metacarpal. This procedure is needed to reinforce the first intermetacarpal ligament after the trapezium excision; at the end, the joint capsule was closed (Fig. 1).

This technique represents a modification of the Epping-Noack technique [10] and of the Burton-Pellegrini technique [14] in which the pedicled half-tendon graft of the FCR tendon is passed through a canal in the first metacarpal base, which is created with a 3.2-mm burr, and sutured to the periosteum of the first metacarpal base. The remainder of the tendon is placed, as a spacer, into the trapezial void and sutured to the FCR tendon.

However, our technique, first described by Altissimi et al. [13], avoids tendon interposition and provides the reinsertion of the radial half of the FCR tendon to the dorsal side of the first metacarpal base using a mini anchor, without any hole in the metacarpal bone. In our patients, after the surgical procedure, the thumb was immobilized in a plaster cast for 2 weeks; after the stitches were removed, a brace was applied, and gradual rehabilitation of the hand was started at the same time.

The patients included in this study came from a pool of 98 patients surgically treated for trapezio-metacarpal osteoarthritis from 2005 to 2017. Fifty-eight patients were excluded from the study because they had undergone another type of surgery (25 patients), or refused to be evaluated (patients lost at follow-up) (29 patients), or were male (4 patients). Considering that trapezio-metacarpal osteoarthritis affects especially women, we preferred to perform the study only on women. We made this decision because the number of operated males was small (8%) and because generally male patients may be heavy manual workers; therefore, in our opinion, including only women made a more homogeneous cohort of patients.

All the patients had severe primary trapezio-metacarpal osteoarthritis corresponding to stage 3 or 4 according to the Eaton-Littler radiological classification [15]. All cases presented marked disability and pain that failed to improve after conservative treatment such as analgesics, anti-inflammatory therapy, splinting, or physiotherapy.

All patients were women, with an average age of 62 years (range 49–74) at the time of operation, and were treated and examined by one or more of the authors.

At follow-up, clinical results were evaluated on the basis of the Disabilities of the arm, shoulder and hand (DASH) score [16], possible presence of pain and the following clinical parameters according to Vermeulen et al. [3]: palmar abduction of the thumb (intermetacarpal distance in millimeters), carpometacarpal joint opposition of the thumb (Kapandji), extension of the metacarpophalangeal joint (degree) and strength of the operated hand compared to the contralateral side (grip strength, tip and key pinch). The DASH outcome measure is a 30-item, self-administered questionnaire designed to assess the patient’s health status [16]. The scores are then used to calculate a scale score ranging from 0 (no disability) to 100 (most severe disability). To evaluate palmar abduction of the thumb, we measured the intermetacarpal distance in millimeters between the first and second metacarpal heads with the thumb in full palmar abduction. In a full palmar abduction, the intermetacarpal distance is about 70 mm [17]. Carpometacarpal joint opposition was evaluated according to the Kapandji scoring system in which score 1 is when the thumb, in maximal opposition, reaches the second finger, while score 10 is when the thumb reaches the distal volar crease of the hand [18, 19]. The metacarpophalangeal joint was evaluated by testing the presence of hyperextension of this joint [3, 18]. The grip strength was measured using a dynamometer, while the tip and key pinch were measured using a pinch gauge [3].

At follow-up, radiographic results were evaluated by a radiographic examination of the hand performed in all patients through standard and oblique views. We evaluated both the presence of proximal metacarpal migration (in millimeters) and carpometacarpal degenerative osteoarthritis.

### Statistical analysis

We performed a descriptive analysis of our study parameters. Data are expressed as mean ± standard deviation. The paired Student’s *t*- test was used to evaluate the significance of the differences between the preoperative and postoperative DASH scores. All statistical analyses were performed using the SigmaStat Version 4.0 program (Systat Software). *P* values less than 0.05 were considered significant.

## Results

**Fig. 2:**
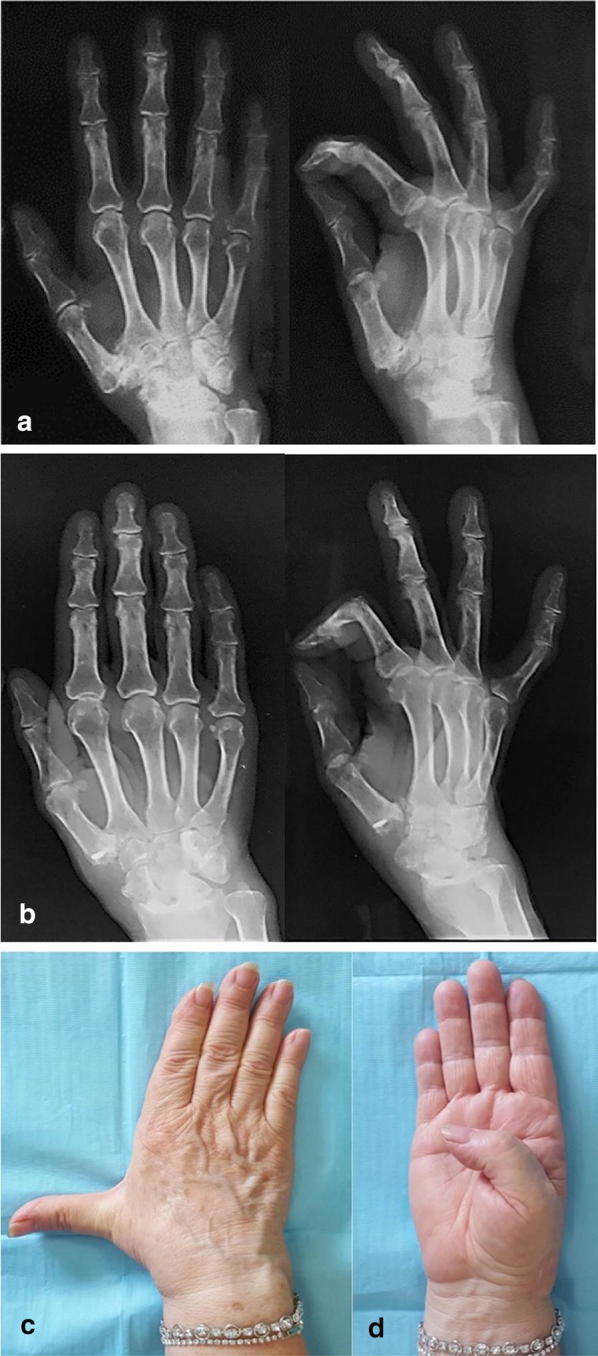
**a** Preoperative radiographs of the right hand showing severe trapezio-metacarpal osteoarthritis in a 57-year-old woman, complicated by pain and disability of the thumb. **b** At follow-up, 9 years after surgery, X-rays did not show proximal migration of the thumb metacarpal or signs of impingement on the scaphoid. **c**, **d** Clinical evaluation showed excellent abduction and joint opposition of the thumb. Metacarpophalangeal hyperextension was evident, as is often observed as a consequence of this surgical procedure. In spite of the hyperextension of the thumb, the patient reported no pain or significant limitations in her daily activities

The average DASH score was 42.65 ± 6.76 (range 35–61) before surgery, whereas it was 16 ± 6.64 (range 0–31) at follow-up, with a statistically significant difference (*P* < 0.001) (Fig. 2).

Thirty-six patients were asymptomatic, while 4 patients reported moderate pain after daily activities.

Palmar abduction of the thumb (intermetacarpal distance) measured from 52 to 67 mm with an average of 57 mm.

Carpometacarpal joint opposition (Kapandji) measured from 6 to 10 with an average of 8.8.

Metacarpophalangeal extension was abnormally increased in 43 thumbs (86%), whereas in only 7 cases was it in the normal range.

The strength of the operated hand was comparable to the contralateral side in 46 cases, while it was moderately reduced in the other 4, especially for tip and key pinch.

Radiographic examinations showed a slight proximal migration of the first metacarpal bone (< 3 mm) in all cases but mild signs of carpometacarpal osteoarthritis in only 4 cases.

## Discussion

Trapezio-metacarpal osteoarthritis is a common form of osteoarthritis of the hand, especially observed in women 50 years old or older. The surgical option is used to treat persistent pain and physical dysfunction of the thumb when conservative treatment fails.

A wide variety of surgical procedures has been advocated for the treatment of carpometacarpal osteoarthritis of the thumb but, to the best of our knowledge, few long-term follow-up studies have been reported. Moreover, it is still debated whether there is a surgical procedure that produces the best results in terms of pain and function of the thumb.

Rizzo et al. [8] published a long-term clinical and radiographic study of 126 trapezio-metacarpal arthrodeses performed in 114 patients (79 women and 35 men) affected by trapezio-metacarpal osteoarthritis. They concluded that, for most patients, trapezio-metacarpal arthrodesis reduces pain, improves function and results in excellent patient satisfaction in spite of the development of metacarpophalangeal and scaphotrapezoid joint arthritis.

In a retrospective study of 107 cases of trapezio-metacarpal arthrodesis, Smeraglia et al. [20] concluded that bone union is not necessary for a good outcome of the surgical procedure.

Epping and Noack [10] reported good results in 97 patients with severe trapezio-metacarpal osteoarthritis treated by trapeziectomy combined with ligament reconstruction and tendon interposition arthroplasty. Good results with their method were also reported by Hilty and Stober [11], Wittemann et al. [12], and Hart et al. [7].

Burton and Pellegrini [14] reported the results obtained in 25 cases of advanced osteoarthritis of the trapezio-metacarpal joint surgically treated by trapeziectomy combined with ligament reconstruction and tendon interposition arthroplasty. Excellent results were achieved in 92% of cases (23 thumbs).

Altissimi et al. [13] reported a personal technique of trapeziectomy and suspension ligamentoplasty with FCR tendon, as a modification of the Burton- Pellegrini procedure [14]. This surgical technique described the same modification which we performed, consisting of avoiding tendon interposition and suturing the radial half of the FCR tendon to the dorsal side of the first metacarpal base using a mini anchor. In their study with a follow-up of 2 to 12 years, the authors conclude that the proposed technique appears effective, simple, minimally invasive, and reliable.

Adams et al. [21] reported the early outcome of spherical ceramic trapezio-metacarpal arthroplasty. They concluded that implant subsidence was often severe, with some resulting in a trapezium fracture, and advised against this kind of implant.

Vitale et al. [22] analyzed 4 types (silicone, Artelon, metal, and pyrocarbon) of trapezium prosthetic arthroplasty, with non-definitive results (“early results have been mixed and further long-term data is required”).

Kriegs-Au et al. [1] compared the long-term outcomes in 43 patients (52 thumbs) treated for primary trapezio-metacarpal osteoarthrosis either by trapezial excision with ligament reconstruction or by trapezial excision with ligament reconstruction combined with tendon interposition. They concluded, with the numbers available, that the amount of proximal metacarpal migration did not differ significantly between the two groups. Furthermore, proximal migration of the first metacarpal does not appear to influence the functional outcome.

Raven et al. [2] reported the long-term results in 54 patients with trapezio-metacarpal osteoarthritis treated by three surgical procedures: resection arthroplasty, trapeziectomy with tendon interposition, and trapezio-metacarpal arthrodesis. They concluded that resection arthroplasty (the joint surfaces of the metacarpal and of the trapezium were resected) is a simple surgical technique and therefore is their preferred technique for the treatment of trapezio-metacarpal osteoarthritis.

Vermeulen et al. [5] published an article with the aim of providing an updated systematic review of the 8 most commonly used surgical procedures to treat trapezio-metacarpal osteoarthritis. They concluded that, at this time, no one surgical procedure is proven to be superior to another.

Similar conclusions were reached by Wajon et al. [6] in a review that included 11 studies with 670 participants and 7 surgical procedures. The authors concluded they were unable to demonstrate that any technique confers a benefit over another technique in terms of pain, physical function, quality of life, and complications.

Vermeulen et al. [3] compared the short-term results obtained in two groups of patients affected by primary trapezio-metacarpal osteoarthritis treated either by trapeziectomy with ligament reconstruction and tendon interposition or by arthrodesis with plate and screws. They concluded that the patients treated by trapeziectomy with ligament reconstruction and tendon interposition have fewer complications in comparison to the patients treated by arthrodesis; the authors do not recommend arthrodesis in women who are 40 years or older. Spekreijse et al. [9] reaffirmed similar results more recently.

In most of our cases, treated by trapeziectomy and ligament reconstruction without tendon interposition according to the modification described by Altissimi et al. [13], we observed very satisfactory functional results at the long-term follow-up. In fact, we observed fair results in only 4 patients, without any poor results.

In 12 thumbs, in spite of reconstruction of the first intermetacarpal ligament by the FCR tendon, we observed, at the final follow-up, a slightly reduced distance between the base of the first metacarpal and the adjacent bones, visible on the radiograph of the hand (proximal migration of the thumb metacarpal). However, no significant functional impairment was observed in most patients and in only 4 cases a slight functional impairment was present, associated with mild signs of carpometacarpal osteoarthritis.

Complete trapezial excision seems to be crucial to obtaining good long-term results; in fact, as long ago as 1949 Gervis [23] reported satisfactory results for treating osteoarthritis of the trapezio-metacarpal joint with excision of the entire trapezium without any soft tissue interposition and without ligament reconstruction.

This simple procedure may cause a proximal migration of the first metacarpal bone, as observed in some of our cases in spite of ligament reconstruction. However, in these cases, functional results were satisfactory without significant loss of strength and stability. Similar data were reported by Krein et al. [24] who did not observe a significant relationship between the height of the arthroplasty space and tip-pinch strength or pain.

The limitations of this study are: first, it is a retrospective study; second, we did not report any control group; third, the patients were only women because we decided to exclude all the males from our cohort of patients. We made this decision because the number of operated males was small (8%) and because generally male patients may be heavy manual workers; therefore, in our opinion, including only women made a more homogeneous cohort of patients.

Based on the reported experience, we conclude that primary trapezio-metacarpal osteoarthritis surgically treated by trapeziectomy and ligament reconstruction without tendon interposition allows good long-term results.

## Data Availability

Not applicable.

## Abbreviations

FCR: Flexor carpi radialis
DASH: Disabilities of the arm, shoulder and hand
